# Facets of Communication: Gap Junction Ultrastructure and Function in Cancer Stem Cells and Tumor Cells

**DOI:** 10.3390/cancers11030288

**Published:** 2019-03-01

**Authors:** Anja Beckmann, Nadine Hainz, Thomas Tschernig, Carola Meier

**Affiliations:** Department of Anatomy and Cell Biology, Saarland University, 66421 Homburg, Germany; Anja.Beckmann@uks.eu (A.B.); Nadine.Hainz@uks.eu (N.H.); Thomas.Tschernig@uks.eu (T.T.)

**Keywords:** cancer, cancer stem cells, gap junctions, connexins, cell-cell communication, ultrastructure

## Abstract

Gap junction proteins are expressed in cancer stem cells and non-stem cancer cells of many tumors. As the morphology and assembly of gap junction channels are crucial for their function in intercellular communication, one focus of our review is to outline the data on gap junction plaque morphology available for cancer cells. Electron microscopic studies and freeze-fracture analyses on gap junction ultrastructure in cancer are summarized. As the presence of gap junctions is relevant in solid tumors, we exemplarily outline their role in glioblastomas and in breast cancer. These were also shown to contain cancer stem cells, which are an essential cause of tumor onset and of tumor transmission into metastases. For these processes, gap junctional communication was shown to be important and thus we summarize, how the expression of gap junction proteins and the resulting communication between cancer stem cells and their surrounding cells contributes to the dissemination of cancer stem cells via blood or lymphatic vessels. Based on their importance for tumors and metastases, future cancer-specific therapies are expected to address gap junction proteins. In turn, gap junctions also seem to contribute to the unattainability of cancer stem cells by certain treatments and might thus contribute to therapeutic resistance.

## 1. Introduction

Worldwide, the majority of deaths are caused by non-communicable diseases. With 9.5 million cancer deaths (non-melanoma skin cancer excluded) estimated by the GLOBOCAN for 2018, cancer is the second leading cause of death worldwide [1,2]. For this reason, understanding the mechanisms behind carcinogenesis is of utmost importance in cancer research and for the development of new therapies. The origination of cancer is caused either by inherited DNA abnormalities or by DNA damage through exposure to mutagenic or carcinogenic substances like chemicals or radiation [3,4,5,6]. The mutations encompass mainly three groups of genes, i.e., proto-oncogenes, tumor suppressor genes and genes for DNA repair [7,8,9,10,11].

Normally, organs and tissues are highly structured consortia of cells, in which expansion ends after completed development. Due to homeostatic regulation, cells then remain either in quiescence or they undergo active modulation like proliferation, differentiation or apoptosis. The control of homeostasis, in turn, is regulated by extracellular signals, and downstream intracellular signals are subsequently transmitted between cells. In consequence, defects in intercellular communication will result in impaired cell homeostasis and is therefore likely to lead to the development of uncontrolled proliferation (growth), namely cancer [12,13]. Cellular communication is granted by different mechanisms, including extracellular vesicles (EV) and tunneling nanotubes (TNT) [14], mainly responsible for long distance contacts and transport of larger components like organelles between cells. The immediate intercellular communication at sites of direct cell contact, in turn, occurs via gap junctions [15]. Gap junctions differ from EVs and TNTs in morphology and function. With the direct connection between two cells and the transport of small molecules (<1 kDa), including water and ions, between cells, gap junctions are a major contributor to the maintenance of cell homeostasis. Thus, the hypothesis arose that gap junctional communication and cancer are interrelated.

In the 1960s, Loewenstein and Kanno were the first to observe the lack of intercellular communication as a key feature of liver tumors: They analyzed the intercellular communication in rat liver cancer and noted the absence of low junctional membrane resistances and the lack of electrical dispersion over distances of many cells as characteristic for normal liver cells [16,17]. From then on, the association of the loss of intercellular communication with cancer initiated many studies, unraveling the role of gap junctions to cancer. Meanwhile, a high number of articles summarizes the involvement of gap junctions in carcinogenesis [18,19,20]. Nevertheless, it is not easy to sum up the role of gap junctions in cancer in a single sentence: For many tumors, malignancy was associated with downregulated connexin expression (Cx43 in prostate [21], pancreatic [22] and breast cancers [23]; Cx26 in colorectal [24] and some gastric cancers [25]). However, malignancy of other tumors was associated with an increased connexin expression (Cx43 in non-muscle invasive urothelial bladder cancer [26]; Cx26 in breast cancer [27], colorectal cancer [28] and papillary and follicular thyroid cancer [29]. Phosphorylation events have been associated with the gain or loss of function as well as with modified localization and plaque size of gap junctions [30,31,32]. Several recent review articles discuss those general aspects in detail [33,34,35].

## 2. Gap Junction Proteins 

**Figure 1:**
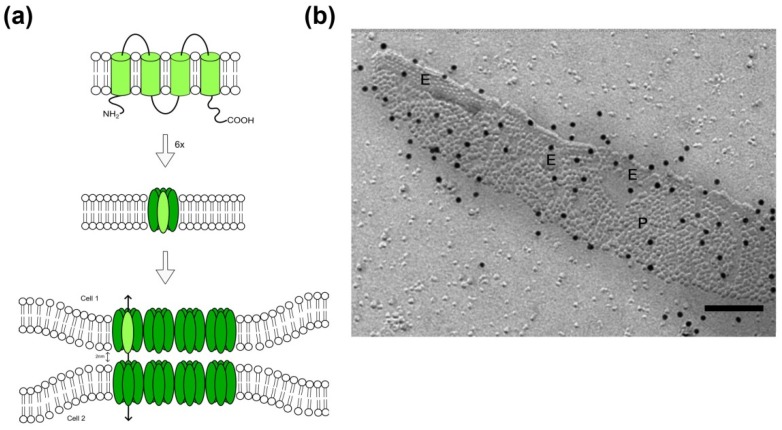
Assembly of connexin proteins to form gap junctions within the plasma membrane of eukaryotes. (**a**) Connexin proteins insert with four transmembrane domains (upper panel; light green) in the plasma membrane of cells. Six connexins oligomerize and form a transmembrane channel (middle panel; hemichannel in green, a single connexin protein is highlighted in light green). Hemichannel-containing membranes of adjacent cells converge to 2 nm, forming an intercellular gap junction. Schematic drawings are not to scale. (**b**) Connexin-gap junction, here shown in the membrane of cultured murine primary astrocytes, display as hexagonally structured plaque in freeze-fracture replicas with particle-rich areas (P-face, P) and areas with pits (E-face, E). Black dots represent Cx43-immunosignal after labeling with 12 nm colloidal gold-coupled secondary antibodies. Scale bar 100 nm.

In the early years of cancer research, the deficiency of electric coupling due to the loss of intercellular communication was identified being an important characteristic for tumor cells [17,36]. Gap junctions are constituents of intercellular communication channels. The protein family of connexins (Cx) with its 21 members in humans known up to now builds the structural basis for the formation of a gap junction plaque: The cytoplasmic N-terminus, four transmembrane domains with two extracellular and one intracellular loop are largely consistent between different connexin proteins. The family members vary mainly in the length of the intracellular C-terminus (Figure 1) [37] and this part of the protein is primarily responsible for variations in the molecular weight of the different connexins. For the formation of membrane channels and subsequently of intercellular gap junctions, two steps are required: At first, six connexin proteins oligomerize into a hexameric structure, called a connexon [15]. These are known to exhibit a function per se, and act as so called hemichannels [38]. Secondly, connexons of two adjacent cells form the cell-cell connecting gap junction, through which small ions and molecules up to 1000 Daltons can pass from one cell to the other [39,40]. Thereby, homotypic (identical connexins in each of the membrane) or heterotypic (different connexins per membrane) gap junctions are formed. Up to several thousand intercellular channels are arranged more or less regularly in the plasma membrane forming gap junction plaques [39,41].

At the ultrastructural level, those gap junctional channel arrangements have a characteristic morphology: In ultrathin sections viewed by transmission electron microscopy, the adjacent membranes display a diminished gap of only 2–3 nm within the gap junctional area, associated with a thickening of these membrane areas [40]. Freeze-fracture allows the plastic view of fractured and replicated membranes, and within these membrane faces gap junctions display as clustered pits or particles, which show a characteristic periodicity (Figure 1). In eukaryotic cells, pits are usually observed in the extraplasmic half of the membrane, named the E-face, whereas particles are found on the protoplasmic membrane leaflet, named P-face. The characteristic morphology of gap junctions allows their identification in freeze-fracture replicas. In a methodological advancement, freeze-fracture replicas were exempted from underlying tissue via SDS digestion, followed by immunolabeling of specific proteins and visualization via colloidal gold-coupled secondary antibodies [42,43,44]. The so-called freeze-fracture replica immunogold labeling (FRIL) technique permits the identification of specific connexins within the gap junction.

**Figure 2:**
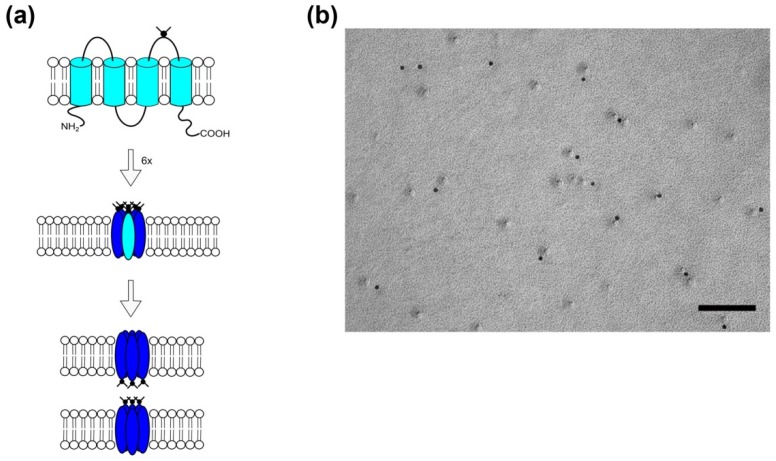
Assembly of Pannexin channels in the plasma membrane of eukaryotes. (**a**) Pannexins insert with four transmembrane domains (lighter blue; top panel) in the plasma membrane of cells. The second extraplasmic loop comprises an N-glycosylation site at residue N254 (black appendage). The pannexin hexamer forms the transmembrane channel (blue; middle panel). The glycosylation is thought to prevent the formation of intercellular pannexin gap junctions (lower panel). Schematic drawings are not to scale. (**b**) Pannexins do not assemble in gap junction-like structured plaques upon over-expression in HEK 293-cells [51]. Shown are freeze-fractured membranes of Panx1-EYFP-transfected cells after immunolabeling using anti-GFP antibodies, detected by 12 nm colloidal gold-coupled secondary antibodies (black dots). Scale bar 100 nm.

In the early 2000s, a family of connexin-related proteins had been identified and named pannexins [45,46], due to their occurrence in vertebrates (and structural similarity to the vertebrate gap junction proteins, the connexins) and their sequence homology to the invertebrate gap junction proteins, the innexins. The pannexin protein family consists of three members, pannexin (Panx) 1 to 3 [47], which are glycoproteins with four transmembrane domains and cytoplasmic C- and N-termini (Figure 2). Panx1 and Panx3 membrane channels are, like connexons, hexameric structures, whereas Panx2 channels seem to assemble as octamers [48]. In contrast to connexins, however, pannexins have not been shown to form intercellular gap junctions under physiological conditions [49,50]. Freeze-fracture analysis of membranes of Panx1-overexpressing HEK cells confirmed the absence of intercellular pannexin channels and, in addition, demonstrated that—in contrast to large gap junction plaques observed in membranes of Cx43 overexpressing cells—pannexin channels would not assemble in plaques [51,52].

Pannexin channels connect the intracellular cytoplasm with the extracellular space, allowing the passage of molecules up to 1.5 kDa, and their opening was shown to be mechanosensitive [50,53,54]. Their main function seems to be that of an ATP-release channel, whereby the extracellular ATP will activate purinergic receptors [55,56,57,58]. Besides the mediation of purinergic signaling, Panx1 plays a role in the immune response by activating the inflammasome and in cellular differentiation and apoptosis [47,59].

## 3. Gap junction Morphology in Carcinogenesis

As aforementioned, the assembly of connexons into a gap junction plaque is crucial for its function as an intercellular channel. Thus, ultrastructural analyses of gap junction morphology in addition to RNA analyses and protein expression studies are important to comprehensively assess gap junctional involvement and its mechanisms in carcinogenesis. The most common ultrastructural approach to study gap junctions is transmission electron microscopy of ultrathin sections after osmiumtetroxid staining or after immunogold labeling. Another approach is freeze-fracture, which displays the ultrastructure of membranes and the integral membrane proteins on single channel level and in a tridimensional manner.

As outlined below, cancerous cells undergo changes in connexin expression and these are likely to be reflected in morphological changes of gap junction plaques. Even in normal cells, gap junctions show a permanent assembly and turnover of particles and are therefore considered highly flexible components in the regulation of physiological processes like cell proliferation and differentiation as well as the maintenance of homeostasis [32,39,60,61]. Accordingly, gap junction morphology is crucial for gap junction function [62]. In view of cancer cells, changes in gap junctions are also very likely to be accompanied by gap junctional plasticity, with phosphorylation and dephosphorylation events being key mechanisms in the modulation of gap junctional disassembly and assembly, respectively. Many connexins have multiple phosphorylation sites for several kinases like the mitogen-activated protein kinases ERK1/2, the protein-kinase C (PKC), the casein kinase 1 (CK1), the serine/threonine-protein kinase Akt or the src protein-tyrosine kinase. Besides the channel opening prevalence, phosphorylation events determine disassembly and internalization of the gap junction plaque: Phosphorylated connexins disband from gap junction plaques, whereas unphosphorylated proteins remain in plaques and provide the means for intercellular gap junction communication [32,61].

The most frequently expressed and therefore most studied connexin is Cx43. Cx43 has a half-life of 1–3 h [63]. Under homeostatic conditions, the dynamic process of assembly and disassembly is a smooth transition: Cx43 is synthesized in the endoplasmic reticulum and is—via the Golgi apparatus path—inserted into the plasma membrane. There, Cx43 connexons are steadied via Zonula occludens-1 (ZO-1) interaction. New connexons, presumably still in a closed state, arrive at the border zone of a gap junction plaque. However, the Cx43-ZO-1 protein interaction itself inhibits the incorporation into the gap junction plaque. This was demonstrated by ZO-1 knockdown studies or studies with a peptide mimicking the Cx43 ZO-1 binding domain [64,65,66].

Thus, the oldest channels assemble in the plaque center, from where they can be internalized for degradation if applicable. Interestingly, this might either occur via endocytosis of membrane segments from one cell (i.e., one membrane) or via internalization of membrane parts of the two connecting cells, resulting in the formation of a double membrane structure containing the gap junction fractions of both cells, so-called annular junctions [67,68]. Phosphorylation on S373 by Akt kinase inhibits the ZO-1 interaction and leads to increased plaque size [69].

The relation of gap junction loss, and with it the loss of cell-cell communication, with abnormal proliferation rates and absence of its regulation in cancer has been demonstrated in many studies [18,19,20]. Here, we would like to outline those studies focusing on the relationship of morphology and cancer. One of the first examples is a study on cervix carcinoma from 1969: An early electron microscopic study demonstrated the absence of any nexus/gap junction in cancer tissue [70]. Similar to this finding in cervix carcinoma cells was the result of an analysis of malignant and benign oncocytic tumors of the thyroid gland [71]. Priollet et al. used the freeze-fracture technique, which displays the ultrastructural assembly of gap junctions at the level of individual connexon. Whereas in samples of normal thyroid tissue from the gland surrounding the tumor, gap junctions appeared inside the tight junctional network, benign oncocytic tumor (adenoma) and malignant oncocytic tumors (carcinoma) lacked identifiable gap junctions. The authors reasoned a gap junction-independent malignancy in case of oncocytic tumors. Interestingly, the structure of tight junction complexes was investigated at the ultrastructural level, too: tight junctions formed a wide-meshed assembly around the apical compartment of normal thyrocytes, evident in freeze-fracture replicas as a belt-like structure of P-face ridges and complementary E-face grooves. In case of the adenoma cells, the tight junctions displayed as relatively normal strands, with the exception of a decreased number of strands in total and an increase of parallel strands. The tight junctions were even organized to a higher degree in oncocytic adenomas and appeared in more parallel strands in oncocytic carcinoma as compared to normal samples. As similar changes in tight junction morphology were also found in poorly differentiated tissues or tumors, this alteration was construed as a sign for a loss of glandular differentiation rather than a tumorigenic factor.

In other cancer types, the number of gap junctions was diminished. Basal cell carcinoma and squamous cell carcinoma showed a lower number of small gap junctions as compared to normal skin and the skin appendages as evidenced by immunoelectron microscopy [72]. The Cx43-immunogoldlabeled particles were distributed all over the cell: Besides their localization to membrane-associated gap junctions, they were found in the cytoplasm in a scattered pattern, possibly pointing to degeneration or internalization of gap junctions. A study on human laryngeal carcinoma demonstrated a noticeable reduction of gap junctions in number as compared to normal larynx epithelia [73].

As these studies were detecting the presence versus absence of gap junctions in normal versus cancerogenic tissue, more detailed morphological observations were required. Morphology was for instance addressed in the ultrastructural assessment of brain tumor gap junctions: In astrocytomas, gap junction morphology changed in that gap junctions were shortened in length and showed deformed nexus [74]. The authors discussed that a self-sustaining network of gap junctions with slight differences in ultrastructure might provide for tumor progression and resistance to therapeutic interventions. In analyses of oligodendroglioma, they noted the absence of any gap junction. A study of Osswald et al. demonstrated the occurrence of Cx43-containing membrane protrusions, called membrane- or nano-tubes, which connect astrocytoma cells [75,76]. Those membrane-tubes are thought to promote cell survival by diluting toxic substances or toxic calcium levels and therefore facilitate the resistance to radiotherapy [75].

In case of meningioma, gap junctions seem to play an important role in the development and malignancy of tumors. A freeze fracture study on human meningioma showed the presence of closed aggregated gap junction particles in fibroblastic and meningiocytic meningioma [77]. Another study presented that typical and benign meningioma showed the same distribution of Cx26 and Cx43. Parallel analyses of protein expression showed a differentiated expression of Cx26 and Cx43 in meningioma subtypes [78]. All benign and atypical meningioma expressed Cx26. For Cx43 in contrast, both phosphorylation forms and to some extent even the unphosphorylated form were expressed in benign meningioma, whereas atypical meningioma expressed only the two phosphoforms and no unphosphorylated protein. Those findings indicate that the gap junction function is reduced in benign forms of meningioma, whereas the gap junction function is diminished in atypical meningioma. The lack of Cx32 and Cx43 protein in hemangiopericytoma seems to be a consequence of its derivation from pericytes compared to the arachnoid cell origin of meningiomas. The lack of connexins was associated with the higher aggressiveness of hemangiopericytoma.

## 4. Gap Junctions in Solid Tumors—Glioblastoma and Breast Cancer Visited Exemplarily

**Figure 3:**
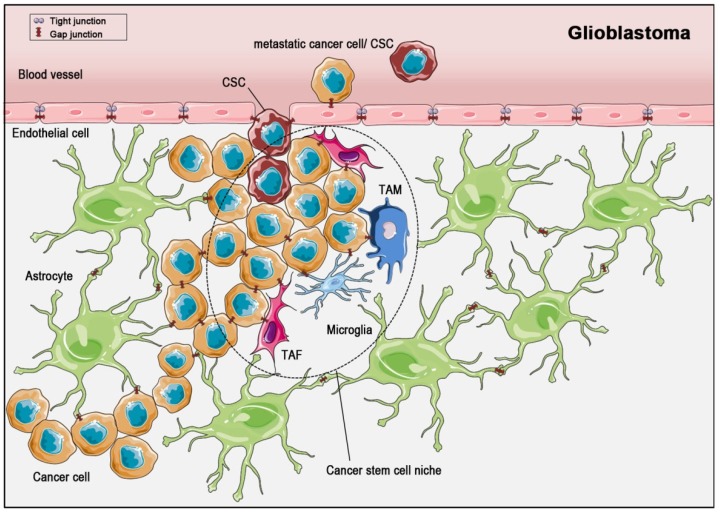
The microenvironment of the glioblastoma stem cell niche. In glioma, cancer stem cells (CSCs) are embedded in a surrounding consisting of tumor-associated macrophages (TAMs), tumor-associated fibroblasts (TAFs), and microglia. Cell-cell communication through gap junctions (red symbols) is known to exist within the astrocytic syncytium, within cancer cell and within CSC populations, as well as between astrocytes and cancer cells, between cancer cells and endothelial cells and between cells of the CSC niche and cancer cells. During metastasis, CSCs and cancer cells develop gap junctions to communicate with endothelial cells, which are normally sealed with tight junctions. Finally, they enter the blood circulatory system for dissemination.

Gliomas are the prevalent form of malignant brain tumors. The incidence for gliomas is 5/100,000 [79] and up to 70% of gliomas are glioblastomas. Gliomas can be astrocytomas, oligodendrogliomas, ependymomas as well as oligo-astrocytomas [79], whereas glioblastomas always derive from astrocytes (Figure 3). Furthermore, glioblastomas can be sub-divided into primary and secondary glioblastomas. The more frequent primary glioblastomas (approximately 85% of all glioblastomas) are very aggressive and develop *de novo*, whereas secondary glioblastomas (around 15% of all glioblastomas) derive from milder precursor astrocytomas [80,81]. The mean survival rate for patients with glioblastoma is 15 months. Glioblastoma cells express Cx43 and Cx30, however, the expression of Cx43 was shown to depend on the stage of tumor cell differentiation [82]. Thus, Cx43 was recently discussed to act as a marker for the prognosis of disease progression and survival [83], particularly as Cx43 expression was shown to differ between therapy-responsive versus therapy-resistant tumors: Munoz et al. showed a significant upregulation of Cx43 mRNA and protein in cells from glioblastoma multiforme patients with resistance to the front-line chemotherapy, temozolomide, as compared to cells from a naïve patient (with temozolomide-sensitive glioblastoma) [84]. For Cx26 and Cx32, only mRNA levels were significantly upregulated in resistant glioblastoma samples compared to samples from untreated glioblastomas, while Cx26 protein was reduced and Cx32 protein was undetectable in resistant glioblastomas. Murphy et al. described resistance to temozolomide in a rat glioma cell line C6 [85]. In a further study, they analyzed Cx43 expression and resistance to temolozomide in samples from patients with glioblastomas. Compared to normal brain tissue, the Cx43 expression was 6- to 14-fold higher in samples from glioblastomas.

O-6-Methylguanine-DNA methyltransferase (MGTM) repair of DNA damage is one mechanism responsible for temozolomide resistance. Thus, glioblastomas were divided into two groups, one group with high MGTM (MGTM^high^) and one group with low MGTM (MGTM^low^) mRNA levels. In the MGTM^high^ group, Cx43 had no effect on survival, whereas in the MGTM^low^ group, patients with high Cx43 levels had a significant shorter survival time compared to low Cx43 levels. In addition, Murphy et al. investigated glioblastoma stem cells and demonstrated high Cx43 levels as well as temozolomide resistance [85]. The link between temozolomide responsiveness and Cx43 was demonstrated in studies employing a selective Cx43-hemichannel inhibitor, which minimized temozolomide resistance. Interestingly, Hitomi et al. showed decreased tumor proliferation in a xenograft mouse model, using a combination therapy of temozolomide and the gap junction inhibitor carbenoxolone [86]. Carbenoxolone is known to inhibit connexin channels as well as pannexin channels [87,88]. Thus, connexin-expression not only changed depending on tumor state and temozolomide-resistance, but conversely, modification of connexin-expression and function by inhibitory agents influenced resistance and tumor cell proliferation, pointing to the putative use of Cx43 in therapeutic approaches.

**Figure 4:**
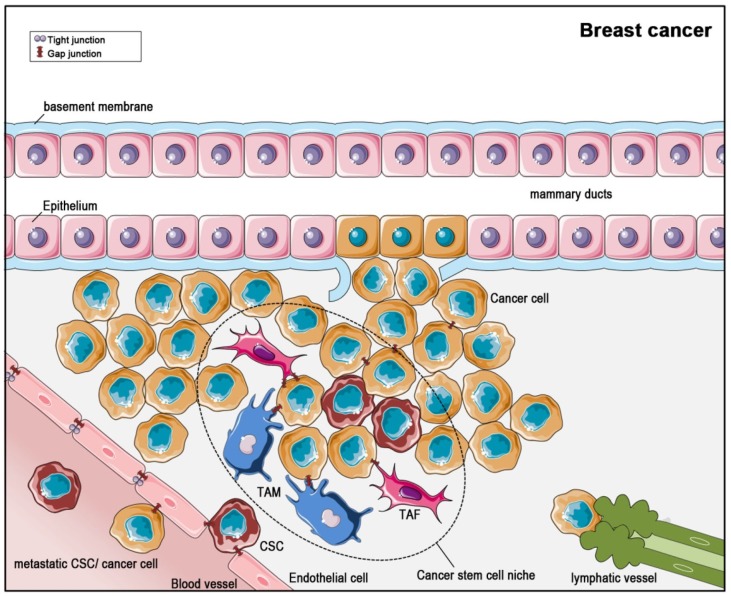
Formation and dispersion of breast cancer with regard to the cancer stem cell niche. Most breast cancers originate from abnormal epithelial cells of the mammary ducts. During tumor progression, the cancer cells break through the epithelial basement membrane. Cancer stem cells (CSCs) settle in a niche of tumor-associated macrophages (TAMs), tumor-associated fibroblasts (TAFs). Gap junction coupling for intercellular communication persist in-between cancer cells, and between the CSC niche cells and cancer cells. In breast cancer, two ways for metastasis exist: In the hematogenic path, cancer cells or CSCs enter the blood circulatory system, initiated by gap junction-mediated communication with endothelial cells. The endothelial cells themselves are sealed by tight junctions and communicate through gap junctions. In the lymphogenic path, CSCs or cancer cells enter lymphatic vessels at their open beginnings.

Breast cancer (Figure 4) is the leading cancer type affecting women all over the world [89]. The survival depends on the stage at which the cancer was diagnosed and differs between industrial countries and less developed countries. The staging ranges from 0 to IV and is based on tumor size, affected tissue areas, surface markers and presence of metastases. In early stages, there is a high chance for cure, but if metastases exist, a criterion for stage IV, there is no effective drug available up to now [90]. Metastasis is not only an important criterion for staging upon first diagnosis. Distant metastases are also found within three years after the initial diagnosis in up to 15% of patients. The fact that even 10 years after first diagnosis micro-metastases are detectable *de novo* indicates that there remains a lifelong risk for metastasis [91].

Several analyses suggest that connexins are involved in metastasis and that connexin expression depends of the stage of cancer: In normal breast tissue, Cx26, Cx30, Cx32, Cx43 and Cx46 were detectable [23] with Cx26 and Cx43 being expressed in cells of the epithelial tree [92]. Lymph nodes from patients with metastasized breast cancer showed higher protein levels of Cx43, Cx26 and Cx32 as compared to primary breast cancer [93]. In a study of 2014, a strong correlation could be found between high connexin levels and improved disease outcome [23]. In 2018, a large-scale microarray analysis on breast cancer tissue conducted last year also revealed a clear association of low Cx43 expression being detrimental for disease outcome with no expression giving the poorest prognosis [83]. In this retrospective study, Cx43 expression profiles of 1118 samples from breast cancer patients were analyzed via a tissue microarray. In about three-quarters of all tumor samples low expression of Cx43 was detected, and this low Cx43 expression was linked to a poor survival prognosis. The distant metastasis-free survival in patients with low Cx43-expression was also worsened. Importantly, Cx43 was claimed to be an independent prognosis factor as the level of Cx43-expression was not related to tumor size, stage or grade but still had a highly significant prognostic value [83].

The data on the role of pannexins in cancer are still quite limited, however, with their function in differentiation, apoptosis and purinergic signaling, a putative role in cancer origination and possibly metastasis seems feasible. There are indeed several reports demonstrating increased levels of Panx1 expression in cancer as compared to non-cancer normal tissue (reviewed by [94]). In most of these studies, many tumors including glioma, melanoma, breast, prostate and colon cancers, were shown to upregulate Panx1 expression ([94] and references within). In contrast, reports of skin cell carcinoma and gall bladder adenocarcinoma state a downregulation of Panx1 expression [95,96].

A first relation between tumor pannexin expression and prognosis was given by Stewart et al. (2016), who studied Panx1 expression and its relevance to disease prognosis in breast cancer. They found that patients with higher Panx1 expression had a poor prognosis for survival, a higher risk for metastases as well as recrudescence compared to patients with lower Panx1 expression [97]. In line with these findings is the recent observation that probenecid, a Panx1 inhibitor, sensitizes breast cancer cells to the treatment with bisphosphonates. Bisphosphonates are frequently used for the treatment of bone metastases, which can for instance derive from breast cancer, kidney cancer and prostate cancer [98].

## 5. Cancer Stem Cells

In recent years, evidence grew that certain stem cells within a tumor were responsible for tumor progression, relapse and the development of metastases [99]. These so-called cancer stem cells (CSCs) are a subpopulation of cancer cells [100]. They are also named “tumor-initiating cells” [101,102] or “tumorigenic cells” [103,104]. CSCs were shown to work similarly to common stem cells in tissue development and regeneration [104]: They have the ability for self-renewal and proliferation, however, in contrast to non-cancer stem cells, they can drive cancer formation [105]. One hypothesis even generalizes their importance to CSCs being the crucial source of tumor initiation, tumor development, metastasis and relapse [99], supported by studies showing that isolation of CSCs and transfer into healthy mice induced solid tumor of the same origin [106]. The population of CSCs, however, is heterogeneous, implying that they have different phenotypes resulting in different functions [99,107].

CSCs were found in several solid tumors, for example breast cancer, colorectal cancer, prostate cancer, and glioblastoma, although they do not seem to occur in all solid tumors [101]. Like non-cancer (normal) stem cells, CSCs settle down in niches [108] (Figure 3 and Figure 4). These niches are distinct areas within the tumor microenvironment, which are characterized by the presence of cancer-associated fibroblasts, endothelial cells, immune cells and others. Interestingly, the production of paracrine factors by stem cell niche-stromal cells stimulates CSCs, induces angiogenesis, recruits immune cells and, last not least, acts positively in a feedback-loop back on the niche-associated stromal cells, producing even more paracrine, tumor-promoting factors [107,108,109]. Thus, the CSCs within the niche are part of a complex network within the tumor and contribute themselves to the creation of a self-strengthening tumor niche [110]. With this complex and finely balanced microenvironment within the niche, CSCs are not only pampered in a tumor-promoting way, but are in consequence also protected from treatments like radiation therapy or chemotherapy. As some tumors are very resistant to therapies and others have a high relapse rate, the CSC-niche is thought to contribute to the extent of response to therapy. Despite pathophysiological characterization, the morphological distinction of cancer cells and CSCs is quite complex [108]: CSCs can be distinguished from other cells using surface markers, but there is not an individual specific CSC marker. However, CD133 expression has often associated to CSC, and in fact, CD133-expression was shown to be a sufficient condition for brain tumor cells to initiate new tumors upon transplantation into NOD-SCID immune-deficient mouse brains [102]. A more comprehensive characterization of CSC surface markers has been performed in breast cancer stem cells, with suggested markers being Bcrp 1, ALDH, CD133, CD176, CD56, CD16, CD44 and CD24 [111].

## 6. Connexins in Cancer Stem Cells

Interestingly, cancer stem cells also express connexins. On the one hand, these connexins seem to be responsible for gap junctional intercellular communication (GJIC) and thus for survival, proliferation and self-renewal of CSCs like they are in other cells [35]. On the other hand, some stem cell-related functions seem to base upon their cytoplasmic localization and interaction with kinases or transcription factors.

When referring to CSCs in terms of a tumor-transmitting cell with self-renewal capacity, irrespective of their pluripotency, the expression of connexins has been demonstrated by many studies and was shown to have a functional impact—despite the connexins themselves possibly being dysfunctional [112]. In general, CSCs depend on intercellular communication, (a) via paracrine secretion of, for example, interleukins, cytokines or pro-angiogenic factors [113,114,115] and (b) via gap junctional intercellular communication. Both modes of cellular interaction are required to maintain stem cell properties, to recruit cells to the stem cell niche (like tumor-associated macrophages (TAMs)), to transform cells in the vicinity of the tumor (like fibroblasts to tumor-associated fibroblasts (TAFs)) or to induce angiogenesis. The function of gap junctions, however, is not yet fully understood, based on their multi-faceted appearance and action: It makes a major difference whether connexin proteins assemble to hemichannels, to intercellular gap junctional channels or whether they remain cytoplasmic proteins. In cancer, all forms have been described. To complicate the picture further, there are major differences between functional roles of connexins in the stem cells of different cancer types. Returning to the examples of breast cancer and glioblastoma, we would like to exemplify these.

The tumor-inducing capability of breast cancer stem cells (BCSC) was identified in 2003 [106]. Meanwhile, it has become evident that breast cancer tumors seemingly have more than one kind of BCSC inside. Identification of cell-surface proteins characteristic for individual BCSCs may lead to the discovery of putative biomarkers. In consequence, identification of a certain BCSC subtype may procure a prognostic outlook. For example, CD44+/CD24- BCSCs are known to be related to a high metastasis rate [116].

In triple-negative (i.e., estrogen receptor, progesterone receptor and HER2/neu receptor-negative) breast cancer, a highly malignant and therapy-resistant form of breast cancer, CSCs show high levels of Cx26 expression [117]. However, the Cx26-function here was demonstrated to be independent of gap junctional communication: Cx26 drove the self-renewal of CSCs via the formation of complexes with the focal adhesion kinase (FAK) and the transcription factor NANOGa [35]. This ternary complex was located in the cytoplasm—with Cx26 executing its function independent of any localization in the plasma membrane. As Cx26 did not co-immunoprecipitate with FAK or NANOG in cells of glandular or ductal tumors, the specific role of Cx26 in triple-negative breast cancer was emphasized. Moreover, transfer of Cx26 into non-CSCs (i.e., MDA-MB-231 and HCC70 cells, which express GFP under the NANOG promotor) resulted in an increase of the pluripotency markers OCT-4 and Sox-2 as well as NANOG itself, the latter being evidenced by GFP-expression [117]. Overexpression of Cx26 was sufficient to induce stem cell properties and self-renewal frequency [117]. The impact of non-communicating Cx26 on the malignant potential of BCSCs allows the expansion of the classical view on connexin function in CSCs. This is in line with a recent review by Trosko, which provides the characterization of cancer stem cells in view of connexin expression: He states that expression of the transcription factor OCT4A, which has been associated with the pluripotency of stem cells [118], is also a characteristic of CSCs, and that those pluripotent CSCs expressing OCT4A do not express connexins and do not form functional gap junctions [112]. In contrast, in those CSCs expressing connexin proteins, these do not form functional gap junctions but serve a communication-independent purpose—which is supported by the data on Cx26 in breast cancer CSCs as outlined above.

In glioblastoma, cancer cells and CSCs also differ in their connexin endowment: The presence of gap junctions was demonstrated in glioblastoma both ultrastructurally and functionally. Further analysis pinpointed Cx46 being expressed in glioblastoma-CSCs and being essential for their proliferation, self-renewal rate and thus tumor initiation capacity [86]. This was in line with high expression levels of Cx46 in glioblastoma CSCs as compared to “non-stem cancer cells” (non-CSCs), in which Cx46 expression was downregulated. Interestingly, Cx43-expression in glioblastoma CSCs and non-CSCs was invers to that of Cx46. According to those findings, Hitomi et al. postulated that Cx46 might be a suitable target to prevent differentiation of CSCs into cancer cells [86]. It is intriguing that the role of Cx46 in glioblastoma-CSCs compares well to that of Cx26 in breast cancer-CSCs. However, in terms of subcellular localization (membrane Cx46 versus cytoplasmic Cx26) and functionality (GJIC via Cx46 channels versus the non-channel function of Cx26), the difference between breast cancer- and glioblastoma-CSCs is supposedly huge. Intriguingly, the study by Yu et al. adds yet another perspective to the existence and function of gap junctions in CSCs: In an *in vitro* system, Yu et al. also showed reduced Cx43 expression in glioma stem cells. This was associated with a higher methylation-status in the Cx43 encoding promotor of gap junction protein α1 in glioma stem cells [119]. In accordance with the findings by Hitomi et al., they also demonstrated that the absence of Cx43 in CSCs was a prerequisite for their self-renewal, invasiveness and tumorigenicity. By reconstituting Cx43 in glioma stem cells (GSCs), self-renewal and tumorigenicity were prevented. These effects coincided with the increased expression of E-cadherin, co-localization with Cx43 and subsequent downregulation of Wnt/ß-catenin target genes. In consequence, Yu et al. reasoned that the upregulation of Cx43 might be a therapeutic target to combat malignant glioma. Furthermore, the mRNA expression levels of additional connexin genes were analysed in glioma stem cells and compared to differentiated cells. The mRNA levels for Cx43, Cx32, Cx50 and Cx26 were upregulated in differentiated cells, while the mRNA levels for Cx46 and Cx30 were higher in glioma stem cells [119]. So far, the data are in line with the studies outlined above. Yu et al. do, however, describe the structural and functional impairment of gap junctions in CSCs, which contrasts the observation of intact and functional gap junctions by Hitomi et al.—and therefore presents a conundrum, for which there is no simple explanation so far. One might postulate that the connexin-set up not only differs between different cancer types but also exhibit regional or cell-type specific variations. This, in turn, would unify the different findings on expression and function of connexins described above.

The low-level expression of Cx43 in glioma stem cells and its importance for their tumorigenicity has been the focus of several studies [119,120,121] and was recently related to the Cx43-mediated inhibition of src [121]. These findings also shed a new light on the association of Cx26, FAK and NANOG described for breast cancer stem cells (see above), as FAK is an important substrate of c-src [122]. Jaraiz-Rodriguez and colleagues pinpointed the responsible region of the Cx43 protein to amino acids (aa) 266-283 and linked the anti-tumor effect to the PTEN-mediated downregulation to glioma stem cell migration and invasion [121].

These studies highlight the importance of Cx43 and its c-src inhibiting sequence (aa 266–283). In view of cell migration being crucial for the dissemination of cancer stem cells as well as non-stem tumor cells, the role of Cx43 in tumor progression as well as therapy is just beginning to be unraveled. As the intriguing link between connexins and metastasis also seems to be relevant for several other tumors (like liver and lung cancer) and for other connexins (like Cx32) [123,124,125,126], targeting connexins in cancer stem cells might provide a promising route for therapeutic approaches. The impact of connexin-expression in CSCs is still being unraveled, but it surely augments their role in common (non-stem) cancer cells. The microenvironment within the stem cells niche seems of equal importance than their functional status including the identity and action of what goes through.

## 7. Cancer Stem Cells, the Stem Cell Niche and Therapeutic Approaches

Resistance to therapy is a hallmark of CSCs and results in the progression of metastasis and relapse. Consequently, CSCs are a prominent target of new drugs. Resistance to therapy can either be acquired as a response to the frequent or long-term use of a drug, or be cell-intrinsic, without any prior contact to the drug. This endogenous resistance was shown to be more common in CSCs than in differentiated cancer cells [127]. CSCs were found in the tumor itself as well as in cells of the tumor niche, a special tissue area contributing to the tumor micro-environment. The tumor-niche does not only consist of CSCs but also of cells of different origin and function, for instance stromal cells, mesenchymal cells, endothelial cells and immune cells. The environment in the niche is crucial for CSC long-term survival. It is noteworthy that all cell types of the niche have independently been described to express connexins. As connexins are known to be important for cell differentiation, cell survival, proliferation and tissue homeostasis, this fact renders the role of gap junction proteins within CSCs and niche-cells very likely.

Unlimited proliferation is one prerequisite for the formation of solid tumors, which, in addition, require the attraction and expansion of blood vessels [128]. The formation of metastases is another characteristic of malignancy. Causing about 90% of the cancer-associated deaths, metastases are the leading cause of death in cancer [129]. To metastasize describes the ability of cancer (stem) cells to spread into distant compartments of the organism and form new tumors. Normal cells (excluding immune cells and bone-marrow derived progenitor cells) without cancer-typical mutations do not show the tendency to invade into other compartments. As outlined above, CSCs are likely to contribute to a large part—if not solely—to the development of metastases. To fulfil their fate as metastasis-mediating cells, CSCs have to leave their niche and migrate through the blood or lymphatic vessel endothelium to distribute. First, they have to immigrate into the vessels (intravasation), then they will be transported to the target tissue site in a lymphogenic or haematogenic manner, and there they have to emigrate (extravasation). Both intravasation and extravasation are promoted by gap junctions, with one major component being endothelial connexin proteins. The interaction between cancer cells and lung endothelial cells was shown to increase upon Cx43 overexpression [130]. Overexpression of Cx43 also fosters formation of brain metastases [131]. In turn, this can be prevented by using RNAi or pharmacologic inhibition to knock-down connexin expression or inhibit connexin channels functionally [34,94]

## 8. Conclusions

Taken studies of the last 40 years of cancer research together, gap junctions clearly participate in the processes of carcinogenesis and cancer. Connexin expression in general is cell-type specific, with one cell type expressing one single connexin (like adult neurons expressing Cx36) or a panel of connexins (like astrocytes expressing Cx43, Cx26 and Cx30). As outlined above, connexin expression was shown to be down-regulated in many cancer types, to the point of complete absence. From this, an anti-tumorigenic effect of connexins has been postulate [35], and this was related to the function and interaction with src [132]. However, other tumors seem to express higher levels of connexins and it is important to focus on whether these connexins are always endogenous or acquired subtypes, which were usually not expressed by this particular cell type. Taken together, defective gap junctions are either a prerequisite for or a consequence of cancer development, and ultrastructural changes, likely to be related to phosphorylation events, are promising targets of future research. Another interesting twist arose from the discovery of cancer stem cells [99,100] and the evolving concept of these cells being responsible for tumor origination, perpetuation and progression in the sense of metastasis. In solid tumors, the invasion stage may be linked to the loss of gap junction function, whereas the metastasis stage might require an increased gap junction function [13]. The involvement of gap junctions seems cancer-specific. At this point, it is important to note that multiple mechanisms are likely to contribute to gap junction action in cancer—and, in addition, gap junction-independent functions of connexins have to be discussed too [133]. In conclusion, connexin and pannexin expression profiles can be used as diagnostic markers for staging cancer and subsequently give a prognosis on cancer progression. With regard to cancer therapy, connexin and pannexin proteins might be suitable targets, (a) in view of their anti-tumorigenic effects, and (b) in view of their ability to overcome therapy resistance of tumor cells.
